# A non-immersive virtual reality-based intervention to enhance lower-extremity motor function and gait in patients with subacute cerebral infarction: A pilot randomized controlled trial with 1-year follow-up

**DOI:** 10.3389/fneur.2022.985700

**Published:** 2022-10-04

**Authors:** Minjie Bian, Yuxian Shen, Yijie Huang, Lishan Wu, Yueyan Wang, Suyue He, Dongfeng Huang, Yurong Mao

**Affiliations:** ^1^Department of Rehabilitation Medicine, The Seventh Affiliated Hospital, Sun Yat-sen University, Shenzhen, China; ^2^Guangdong Engineering and Technology Research Center for Rehabilitation Medicine and Translation, Guangzhou, China

**Keywords:** virtual reality, ischemic stroke, gait analysis, motor activity, rehabilitation

## Abstract

**Introduction:**

This study was conducted to evaluate whether a non-immersive virtual reality (VR)-based intervention can enhance lower extremity movement in patients with cerebral infarction and whether it has greater short-term and long-term effectiveness than conventional therapies (CTs).

**Materials and methods:**

This was a single-blinded, randomized clinical controlled trial. Forty-four patients with subacute cerebral infarction were randomly allocated to the VR or CT group. All intervention sessions were delivered in the inpatient unit for 3 weeks. Outcomes were measured before (baseline) and after the interventions and at 3-month, 6-month and 1-year follow-ups. The outcomes included clinical assessments of movement and balance function using the Fugl–Meyer Assessment of Lower Extremity (FMA-LE) and Berg Balance Scale (BBS), and gait parameters in the sagittal plane.

**Results:**

In the VR group, the walking speed after intervention, at 3-month, 6-month, and 1-year follow-ups were significantly greater than baseline (*p* = 0.01, <0.001, 0.007, and <0.001, respectively). Compared with baseline, BBS scores after intervention, at 3-month, 6-month, and 1-year follow-ups were significantly greater in both the VR group (*p* = 0.006, 0.002, <0.001, and <0.001, respectively) and CT group (*p* = <0.001, 0.002, 0.001, and <0.001, respectively), while FMA-LE scores after intervention, at 3-month, 6-month, and 1-year follow-ups were significant increased in the VR group (*p* = 0.03, <0.001, 0.003, and <0.001, respectively), and at 3-month, 6-month, and 1-year follow-ups in the CT group (*p* = 0.02, 0.004 and <0.001, respectively). In the VR group, the maximum knee joint angle in the sagittal plane enhanced significantly at 6-month follow-up from that at baseline (*p* = 0.04).

**Conclusion:**

The effectiveness of the non-immersive VR-based intervention in our study was observed after the intervention and at the follow-ups, but it was not significantly different from that of CTs. In sum, our results suggest that non-immersive VR-based interventions may thus be a valuable addition to conventional physical therapies to enhance treatment efficacy.

**Clinical trial registration:**

http://www.chictr.org.cn/showproj.aspx?proj=10541, ChiCTR-IOC-15006064.

## Introduction

Stroke is a major health problem with a global incidence of almost 12.2 million cases each year, and has been identified as the third-leading cause of both death and disability in recent years (1). In China, stroke incidence has almost doubled over the past 30 years, posing a great burden to Chinese society, and the ischemic stroke accounted for more than 80% (2). Approximately 88% of post-stroke patients discharged from hospitals continue to suffer from impaired walking ability (3). Moreover, ~50% of post-stroke patients who regain ambulation capability continue to experience difficulties in walking in the community (4). Limited walking ability is a major concern for stroke survivors, both physically and psychologically, as it has a negative impact on their daily function and, ultimately, their quality of life due to limited access to the community (5). The gait pattern among post-stroke survivors usually shows lower walking speed, and an abnormal hip-knee-ankle joint movement (6). Therefore, gait recovery is a major objective of stroke rehabilitation.

To improve gait function in post-stroke patients, continued physical therapy at all recovery stages is necessary. Therapeutic techniques such as virtual reality (VR) are being increasingly applied in neurorehabilitation practice, and the benefits of applying VR-based training have been widely recognized in the field of stroke rehabilitation. VR technique can be divided into non-immersive VR with different levels of immersion and immersive VR with a head-mounted display, which is closer to real-life but is easy to leave the adverse effect of dizziness (7). The effectiveness of immersive VR has been demonstrated to train motor patterns of healthy young participants, and the patterns were maintained in real-world settings (8). However, in China, the non-immersive VR is relatively not expensive and user-friendly for both therapists and patients, which is commonly applied in rehabilitation therapy. Hence this study utilized the non-immersive VR techniques to clarify the effectiveness of VR technique. VR is an advanced computer–human interface that provides artificial sensory feedback for patients while they perform real-time tasks and experience real-time events in virtual environments (9). Training with VR is considered to include the rehabilitation principles of high-intensity, repetitive and task-specific practice (10). Moreover, VR is well-recognized to improve motivation and enjoyment and consequently decrease the perception of exertion, which promotes adherence to the training activity (5). In addition, VR could reinforce the physiological basis of motor learning and descending neural pathways (11, 12), and its potential cognitive benefits to patients, including improvements in attention or memory, have been demonstrated in situations where they are required to react quickly and deal with busy environments with multiple stimuli. Therefore, thus, VR could play a beneficial role in improving balance and gait capacity among post-stroke patients (13). In clinical, VR is applied independently or in combination with the abovementioned conventional physical therapy techniques for gait rehabilitation and motor function improvement.

Recently, studies on the effectiveness of non-immersive VR among post-stroke patients have reported inconsistent findings. Some reviews have reported that the current evidence is insufficient to conclude that VR is more effective than conventional therapy (CT) (10, 14–16), while others suggested that VR enhances the lower-limb motor performance including balance and gait function of post-stroke patients more efficiently than CT (4, 17–21). Specifically, several RCTs showed no statistical differences between the effects of VR treatment and CT treatment on balance or lower extremity motor function for acute stroke patients and subacute patients (22, 23). On the other hand, one study has demonstrated that non-immersive VR as an add on to CT was more effective in balance capability than CT alone among subacute patients without long-term result reported (24). Several studies that adopted clinical measures to assess lower extremity and gait ability have also suggested VR's potential to promote functional recovery for chronic stroke patients (25), while some have shown the benefits of VR in enhancing balance capability (26, 27). Moreover, an RCT showed that cycling training with smartphone VR application led to significant improvements in lower extremity function, sitting balance and spatiotemporal gait performance for chronic stroke patients compared with CT (12). The controversial findings for the motor function after VR intervention possibly result from insufficient VR programs designed for impairments, and the largely varied duration before intervention (16). VR combined with conventional physiotherapy contributed to motor improvement in post-stroke patients in both subacute and chronic stages, but improvement of kinematic outcomes was confirmed for the subacute group, but not for the chronic group (28). Therefore, in our study, different VR programs with the same dosage were applied for lower-extremity motor function and gait, and subacute post-stroke patients were recruited.

Moreover, the majority of published studies followed patients for no longer than 3 months and reported contradictory follow-up outcomes. Wii-based VR intervention was found to increase Berg Balance Scale (BBS) scores both immediately after the intervention and at the 4-week follow-up, and that the scores at both time points were greater than those of the CT group (29). Nevertheless, another RCT concluded that the effectiveness of VR on balance and gait could not be maintained, and found that the CT group showed greater improvements in weight-bearing symmetry at the 3-month follow-up than the exergame group, among chronic and subacute stroke patients (30). Similarly, the effect of non-immerisve VR was shown not statistically different from that of CTs in improving balance performance or gait capability at both post-intervention and the 3-month follow-up among chronic and subacute stroke patients (23, 31, 32). Furthermore, one systematic review summarized that the evidence on the effectiveness of VR analyzed using biomechanical parameters was limited, especially for sustained effectiveness at longer than 4 weeks post-intervention (17). Additionally, the effectiveness of treatment in the follow-ups contribute to the evidence of motor relearning and neural plasticity, which is significant for stroke patients (33).

Therefore, the current study evaluated the long-term effects of a VR-based intervention, i.e., at 6 months and 1 year post-intervention, using biomechanical analyses of lower extremity motor function, balance function and gait pattern.

Given the above-mentioned contradictory results and no reported long-term follow-up result, our study aimed to clarify whether a 3-week course of VR-based lower extremity exercises can effectively improve gait parameters and motor function in post-stroke inpatients in the subacute stage, and whether the effects of these exercises on motor function and gait are sustained at a 1-year follow-up.

## Materials and methods

### Study participants

Inpatients in the subacute phase after cerebral infarction stroke were recruited from the Seventh Affiliated Hospital and the First Affiliated Hospital, Sun Yat-Sen University, China. Adults without neurological pathology were recruited through advertisement. This study was approved by the Human Subjects Ethics Subcommittee of the Affiliated Hospitals (NO.2019SYSUSH-019), Sun Yat-Sen University, China. Clinical trial registration number is ChiCTR-IOC-15006064. Written consent was obtained from all participants prior to inclusion in the study. The study was conducted from October 2019 to June 2022.

Potential post-stroke inpatients were recruited by physicians at the rehabilitation center using the following inclusion criteria: post-stroke inpatients with (1) Mini-Mental State Examination scores >24 (34); (2) National Institute of Health Stroke Scale scores <20 to exclude heavy ischemic stroke (35); (3) diagnosis of cortical and subcortical schemic stroke (confirmed by magnetic resonance imaging or computer tomography) <6 months before their inclusion in this study; (4) Brunnstrom stage for lower-extremity ≥1; (5) no previous VR-based rehabilitation training experience; (6) Modified Ashworth Scale scores of the lower-limbs ≤ 2; (7) the ability to maintain sitting balance for more than 20 min and walking for over 10 meters; and (8) adults under the age of 75. The exclusion criteria were (1) patients who had previously received VR-based training, and (2) patients with other diseases, such as cerebellar and brainstem injuries, severe cognitive impairment, joint stiffness, convulsive crisis, congestive heart failure, deep vein thrombosis of a lower extremity, malignant progressive hypertension, respiratory failure, active liver disease, severe hepatic and renal insufficiency, history of mental illness and inability to cooperate. Normal age-matched adults were included, if they have no previous and current central nervous system diseases and severe musculoskeletal diseases.

### Study design

The study was a single-blinded, randomized controlled trial to explore the effects of VR gait training on the motor function of patients with subacute cerebral infarction. All participants were randomly allocated to either a VR group or a CT group. The baseline and post-intervention assessments and training sessions were conducted in the hospital's inpatient rehabilitation department, and post-intervention and 3-month, 6-month, and 1-year follow-up assessments were performed in the outpatient rehabilitation center. Sample size estimation was performed by G^*^Power software (Düsseldorf, Germany), considering a Minimal Clinically Important Difference (MCID) change of walking speed equal to 0.13 m/s (36, 37) as the expected effect of the treatment, with a pooled Standard Deviation (SD) of 0.23. Thus, for α = 0.05, β = 0.2, with an f effect size = 0.57, it provides an estimated total sample size of 42 subjects. Moreover, considering a 10% dropout, 47 total subjects were considered sufficient for statistical analysis.

An independent researcher conducted the random allocation of participants based on a randomization sequence generated by a statistics expert from Sun Yat-sen University. Allocation numbers were sealed in opaque envelopes. The researchers performing the assessments were blinded to treatment allocation, but the participants and the therapists providing the interventions could not be blinded due to the nature of the interventions.

### Intervention

**Figure 1:**
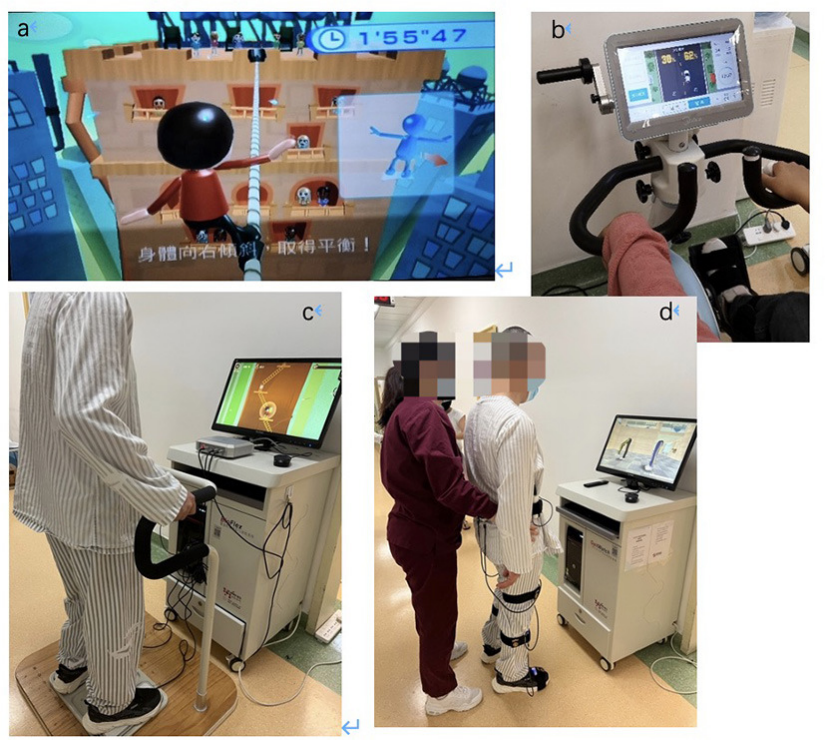
Examples of VR interventions. **(a)** Wii exergame training; **(b)** an active and passive trainer with a VR screen; **(c)** a VR balance training system; **(d)** lower extremity training in a VR gait training system.

The interventions in this study mainly focused on the functional ability of the lower extremities, including lower limb movement, balance training and gait exercise. All participants received 5-h rehabilitation programs (either VR-based or CT) on 5 days per week for 3 weeks. Specifically, the VR group received 15 physical training sessions combined with VR-based training, while the CT group underwent routine CT-based rehabilitation training for the same duration. In each session of the two programs, the training intensity was adjusted by experienced physical therapists in line with the participants' progress, safety and movement quality. VR techniques adopted in the VR group included the Wii exergame training system, an active and passive trainer with a VR screen, a VR balance training system and a VR gait training system based on the non-immersive VR techniques with feedback including visual, auditory, and numbers. The examples of them are shown in Figure 1 (trajectory tracking, car driving and etc.) and each VR training system is detailly descripted in the Table 1. Therapists introduced, demonstrated and guided the patients at the first time of VR intervention, they also supervised the intervention of training of patients during the following sessions. The difficulty level of VR training was modified by experienced therapists based on the abilities and therapeutic goals of each participant, and the system displayed the outcome of each VR training session for the participants and therapists once the training ended.

**Table 1:** VR training systems applied in the study.

**Catagories**	**Sensor used**	**Movement executed**
Wii exergame training system	Camera	Balance training (dynamic)
An active and passive trainer with a VR screen	Sensors (speed and moment)	Cycling
A VR balance training system	Pressure transducers	Balance training (standing)
A VR gait training system	Sensors (speed and direction)	Walking, stepping and lower extremity training

### Outcome measures

**Figure 2:**
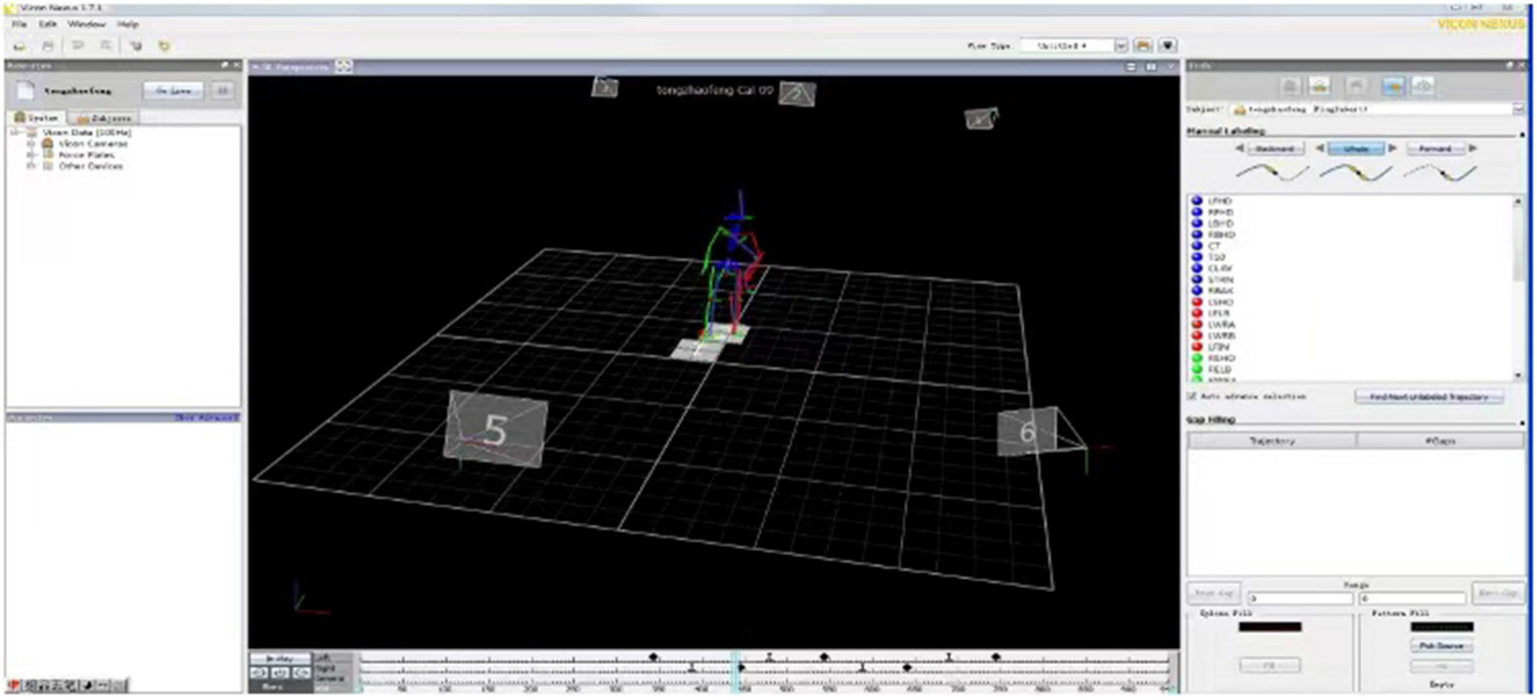
Three-dimensional data capture.

The outcomes of the intervention participants were measured before and immediately after the 3-week intervention and at 3-month, 6-month, and 1-year follow-ups using standard operating procedures. The collected data included demographic data (i.e., age, gender, affected side), clinical assessment outcomes of National Institute of Health stroke scale (NIHSS), Mini Mental State Examination (MMSE), Fugl-Meyer assessment (FMA-LE) and BBS, as well as biomechanical parameters recorded using a 3D gait analysis system, the walking speed parameter of which is the primary outcome of this study. Intention-to-treat analyses were done during the data analysis. The gait performance of twelve age-matched healthy adults were also assessed for comparison. All biomechanical data on gait was obtained and analyzed using a real-time motion tracking/capture system, namely, the standard PlugInGait model with Vicon Nexus software (version 1.7.1; Vicon Motion Systems, UK), as shown in Figure 2. Six infrared 100-Hz cameras recorded the location of 16 markers of pelvic and both lower-extremities during the data collection under the guidance of model sets of PlugInGait model, including the midline sacrum at the level of the posterior superior iliac spines, anterior superior iliac spines, lower lateral 1/3 and 1/2 surface of left and right thigh, lateral epicondyle of knee, lower lateral 1/3 and 1/2 surface of left and right shank, lateral malleolus, the second metatarsal head, and the calcaneus at the same height as the toe marker. The spatiotemporal and lower-extremity joint kinematic data was obtained from the model. During the gait assessment process, each participant was asked to wear flat shoes and close-fitting pants and to walk independently for 10 meters without any crutches or ankle foot orthoses, turn around and return to the starting point at a self-selected walking speed. One researcher collected gait data and calculate the number of successful gait cycles for minimum of six. In addition, during clinical outcome assessments, the post-stroke participants' FMA-LE (38) and BBS scores for lower limb performance were obtained (39, 40).

### Data processing and analysis

Gait data from successful gait cycles, specifically the spatiotemporal and kinematic parameters, were analyzed using Polygon (version 3.5.1; Vicon Motion Systems, UK). Heel strike events from each walking trial were determined by visual inspection, with reference to the Vicon Nexus software output, and were used to define the beginning and end of each walking cycle. The following spatiotemporal parameters were collected and analyzed: cadence, stride time, opposite foot off, opposite foot contact, step time, single support, double support, opposite foot off, stride length, step length and walking speed.

Additionally, the angles of the hip, knee and ankle joints of the affected lower limbs were measured. Subsequently, from the mean profile of selected strides, data on the following joint kinematic parameters in the sagittal plane were extracted: the maximum angle, minimum angle and range of motion for the hip, knee and ankle joints. For comparison, these joint kinematic parameters from the gait data of 12 adults without neurological pathology were also analyzed.

Statistical analysis was performed using GraphPad Prism version 9.0.0 for macOS (GraphPad Software, San Diego, California USA). The normality of data on parameter was checked using the Shapiro–Wilk test and a normal Q–Q plot, and the normality of each outcome was confirmed. Unpaired *t*-test and X2 test were used to compare between-group differences at baseline for continuous variables and for categorical variables, respectively. Repeated-measures two-way ANOVA was used for detection of within-group differences from pre-intervention to post-intervention to follow-ups, while a *post-hoc* analysis with Bonferroni's correction was used for between-group differences at all time points.

## Results

Forty-four participants with post-stroke hemiparesis were included in this pilot study and randomly allocated to the VR or CT group. According to the randomization sequence, 23 participants were allocated to the VR group and 21 to the CT group. At the 3-month follow-up, the number of participants in the VR and CT groups has been decreased to 17 and 19, respectively. At the 6-month and 1-year follow-ups, the number of participants in the VR and CT groups was 16 and 18, respectively. The outcome of those 16 and 18 participants in the VR and CT groups were analyzed. The demographic characteristics of the participants, the outcomes of clinical scales and the walking speed are presented in Table 2 and were not significantly different between the two groups.

**Table 2:** Demographic characteristics (Mean ± SD) at baseline for three groups.

	**Normal**	**VR group**	**CT group**	***P*-value**
Age	58.17 ± 8.12	53.25 ± 8.72	55.00 ± 10.27	0.599
Gender (male/female)	6/6	14/4	13/3	0.803
Affected side (right/left)		10/8	6/10	0.292
NIHSS		4.69 ± 2.50	4.56 ± 2.854	0.888
MMSE		28.56 ± 1.83	28.06 ± 1.73	0.412
BBS		36.94 ± 10.55	38.28 ± 14.05	0.758
FMA-LE		23.10 ± 5.89	23.50 ± 4.46	0.834
Walking speed		0.42 ± 0.20	0.43 ± 0.21	0.812

MMSE, Mini Mental State Examination; NIHSS, National Institute of Health Stroke Scale; BBS, Berg Balance Scale; FMA-LE, Fugl-Meyer Assessment-Lower Extremity.

### Balance and lower limb motor function

Compared with the BBS scores at baseline, those after intervention, at 3-month, 6-month, and 1-year follow-ups were significantly greater in both the VR group (*p* = 0.006, 0.002, <0.001, and <0.001, respectively) and CT group (*p* ≥ 0.001, 0.002, 0.001, and <0.001, respectively) (Table 3). Meanwhile, compared with the FMA-LE scores at baseline, those after intervention, at 3-month, 6-month, and 1-year follow-ups were significant increased in the VR group (*p* = 0.03, <0.001, 0.003, and <0.001, respectively), and at 3-month, 6-month, and 1-year follow-ups in the CT group (*p* = 0.02, 0.004, and <0.001, respectively). Notably, both the BBS and FMA-LE scores before the intervention, immediately post-intervention and at the follow-ups were not significantly different between the VR and CT groups.

**Table 3:** Balance and lower motor function results of pre, post and follow-ups between CT and VR group.

**Variable**	**VR group**					**CT group**				
	**Baseline**	**Post**	**3 m** **follow-up**	**6 m** **follow-up**	**1 yr** **follow-up**	**Baseline**	**Post**	**3 m** **follow-up**	**6 m** **follow-up**	**1 yr** **follow-up**
BBS	36.94 ± 10.55	38.38 ± 8.72*	48.88 ± 5.74*	52.00 ± 4.66*	53.00 ± 3.74*	38.28 ± 14.05	35.86 ± 13.15*	43.00 ± 10.47*	44.71 ± 11.25*	47.00 ± 10.42*
FMA-LE	23.10 ± 5.89	21.63 ± 5.55*	25.88 ± 5.06*	27.13 ± 4.29*	27.50 ± 4.07*	23.50 ± 4.46	23.00 ± 5.35	24.14 ± 5.93*	25.43 ± 5.26*	25.71 ± 4.92*

^*^Significant difference between pre and follow-ups of intervention (P <0.05).

### Spatiotemporal gait parameters

In the VR group, the walking speed after intervention, at 3-month, 6-month, and 1-year follow-ups were significantly greater than baseline (*p* = 0.01, <0.001, 0.007, and <0.001, respectively); additionally, cadence at 3-month, 6-month, and 1-year follow-ups were significantly greater than baseline (*p* = 0.002, 0.02, and <0.001, respectively). Stride time at 3-month and 1-year follow-ups and opposite foot contact at 6-month follow-ups were significantly greater than baseline in the VR group (*p* = 0.04 and 0.002, respectively). In addition, the decrease of step time (*p* = 0.03, 0.01, 0.007, and 0.008, respectively) and the increase of stride length (*p* = 0.003, 0.002, 0.002, and <0.001, respectively) after intervention, at 3-month, 6-month, and 1-year follow-ups changed significantly from baseline, while the decrease of double support (*p* = 0.006, 0.01, and 0.01, respectively) and the increase of step length (*p* = 0.009, 0.02, and <0.001, respectively) at 3-month, 6-month and 1-year follow-ups differed significantly from baseline. No significant change was found in the CT group. The outcomes of all spatiotemporal parameters were not significantly different between the VR and CT groups at all time points. All spatiotemporal gait data are provided in Table 4.

**Table 4:** Spatiotemporal results of pre, post and follow-up among three groups.

**Variable**	**Normal**	**VR group**					**CT group**				
		**Pre**	**Post**	**3 m follow-up**	**6 m follow-up**	**1 yr follow-up**	**Pre**	**Post**	**3 m follow-up**	**6 m follow-up**	**1 yr follow-up**
Walking speed	0.96 ± 0.13	0.42 ± 0.21	0.52 ± 0.24*	0.65 ± 0.28*	0.65 ± 0.27*	0.73 ± 0.24*	0.43 ± 0.21	0.50 ± 0.24	0.55 ± 0.28	0.57 ± 0.24	0.62 ± 0.23
Cadence	108.13 ± 10.65	74.63 ± 16.06	79.35 ± 18.16*	87.78 ± 16.34*	88.05 ± 13.01*	93.36 ± 14.53*	72.62 ± 24.12	77.21 ± 27.67	77.07 ± 22.40	77.78 ± 20.05	82.09 ± 17.52
Stride time	1.12 ± 0.11	1.70 ± 0.43	1.62 ± 0.42	1.42 ± 0.28*	1.40 ± 0.21	1.32 ± 0.22*	1.84 ± 0.63	1.76 ± 0.65	1.70 ± 0.55	1.66 ± 0.49	1.56 ± 0.48
Opposite foot off	11.14 ± 1.35	15.88 ± 4.93	15.61 ± 7.25	12.52 ± 4.97	12.68 ± 3.35	12.61 ± 3.04	18.60 ± 12.00	18.91 ± 0.80	16.49 ± 10.53	15.46 ± 9.07	20.34 ± 20.41
Opposite foot contact	49.60 ± 1.36	44.57 ± 6.07	47.21 ± 5.39	47.63 ± 3.65	49.28 ± 5.48*	46.70 ± 3.34	45.94 ± 6.12	45.65 ± 4.72	48.96 ± 6.38	46.62 ± 6.95	45.41 ± 7.78
Step time	0.56 ± 0.06	0.95 ± 0.30	0.86 ± 0.25*	0.75 ± 0.17*	0.71 ± 0.14*	0.71 ± 0.15*	1.01 ± 0.41	0.98 ± 0.43	0.86 ± 0.26	0.90 ± 0.35	0.87 ± 0.37
Single support	0.43 ± 0.04	0.47 ± 0.11	0.49 ± 0.09	0.49 ± 0.09	0.51 ± 0.10	0.44 ± 0.06	0.50 ± 0.08	0.44 ± 0.14	0.52 ± 0.10	0.49 ± 0.07	0.37 ± 0.35
Double support	0.25 ± 0.04	0.61 ± 0.29	0.54 ± 0.31	0.38 ± 0.19*	0.37 ± 0.12*	0.38 ± 0.16*	0.67 ± 0.50	0.73 ± 0.50	0.57 ± 0.42	0.55 ± 0.35	0.58 ± 0.42
Foot off	60.82 ± 1.90	63.54 ± 5.80	63.29 ± 6.09	61.25 ± 4.57	62.78 ± 5.29	62.27 ± 4.27	62.63 ± 7.71	65.45 ± 6.38	62.78 ± 5.20	62.18 ± 5.04	61.00 ± 5.53
Stride length	1.06 ± 0.11	0.65 ± 0.20	0.76 ± 0.21*	0.85 ± 0.25*	0.87 ± 0.28*	0.93 ± 0.20*	0.69 ± 0.20	0.75 ± 0.22	0.81 ± 0.28	0.84 ± 0.23	0.88 ± 0.22
Step length	0.54 ± 0.07	0.33 ± 0.11	0.38 ± 0.11	0.44 ± 0.11*	0.44 ± 0.13*	0.48 ± 0.10*	0.38 ± 0.11	0.38 ± 0.11	0.43 ± 0.11	0.43 ± 0.12	0.46 ± 0.11

^*^Significant difference between pre and follow-ups of intervention (P <0.05).

### Kinematic parameters

In the VR group, the maximum knee joint angle in the sagittal plane enhanced significantly at 6-month follow-up from that at baseline (*p* = 0.04) and no significant difference was found in any other parameter. In the CT group, no significant difference was found between baseline and other time points. Moreover, all kinematic parameters were not significantly different between the VR and CT groups at all time points. All kinematic gait parameters are provided in Table 5.

**Table 5:** Kinematic results in the sagittal plane of pre, post and follow-up among three groups.

**Variable**	**Normal**	**VR group**					**CT group**				
		**Pre**	**Post**	**3 m follow-up**	**6 m follow-up**	**1 yr follow-up**	**Pre**	**Post**	**3 m follow-up**	**6 m follow-up**	**1 yr follow-up**
Hip-max	30.20 ± 7.85	20.85 ± 7.02	24.80 ± 6.18	21.26 ± 11.17	24.82 ± 10.43	20.91 ± 10.07	20.94 ± 8.77	21.90 ± 9.57	19.55 ± 10.39	21.08 ± 9.81	22.14 ± 9.88
Knee-max	57.28 ± 10.36	29.82 ± 16.14	36.04 ± 14.62	38.07 ± 14.63	43.87 ± 21.34*	44.43 ± 21.62	32.67 ± 15.61	36.50 ± 17.08	31.85 ± 16.30	31.66 ± 18.29	37.84 ± 18.75
Ankle-max	9.84 ± 7.14	13.41 ± 3.82	13.61 ± 6.03	12.91 ± 8.67	13.77 ± 2.11	11.36 ± 3.66	11.95 ± 7.82	12.11 ± 6.51	12.74 ± 6.56	9.82 ± 3.91	12.77 ± 4.21
Hip-min	−8.56 ± 7.49	−7.21 ± 9.79	−6.75 ± 11.51	−11.09 ± 16.51	−8.53 ± 12.72	−13.51 ± 1.25	−6.75 ± 9.91	−8.65 ± 13.23	−11.37 ± 8.68	−9.29 ± 9.95	−10.04 ± 7.72
Knee-min	5.63 ± 4.67	2.65 ± 7.53	4.00 ± 7.77	0.83 ± 9.09	2.90 ± 5.56	0.75 ± 6.14	−0.71 ± 8.81	0.53 ± 8.64	−1.31 ± 1.71	−3.24 ± 7.39	−1.88 ± 7.58
Ankle-min	−16.47 ± 7.21	−5.36 ± 5.93	−6.91 ± 8.80	−6.28 ± 8.49	−4.63 ± 5.63	−8.84 ± 5.85	−9.67 ± 7.96	−8.19 ± 6.73	−10.22 ± 5.02	−9.49 ± 5.11	−8.75 ± 6.10
Hip-range	38.76 ± 3.68	28.05 ± 8.68	31.56 ± 10.95	32.35 ± 8.89	33.35 ± 11.28	34.42 ± 7.4	27.69 ± 8.10	30.55 ± 8.98	30.92 ± 10.83	30.37 ± 11.61	32.18 ± 10.57
Knee-range	51.65 ± 8.45	27.17 ± 14.48	32.05 ± 15.20	37.24 ± 16.82	40.97 ± 20.46	43.68 ± 18.82	33.38 ± 12.33	35.96 ± 14.06	33.15 ± 14.74	34.91 ± 6.98	39.72 ± 16.71
Ankle-range	26.30 ± 5.84	18.77 ± 6.59	20.52 ± 7.39	19.19 ± 5.02	18.40 ± 6.30	20.20 ± 6.75	21.62 ± 9.07	20.30 ± 6.23	22.96 ± 6.95	19.31 ± 3.13	21.52 ± 4.57

^*^Significant difference between pre and follow-ups of intervention (P <0.05).

**Figure 3:**
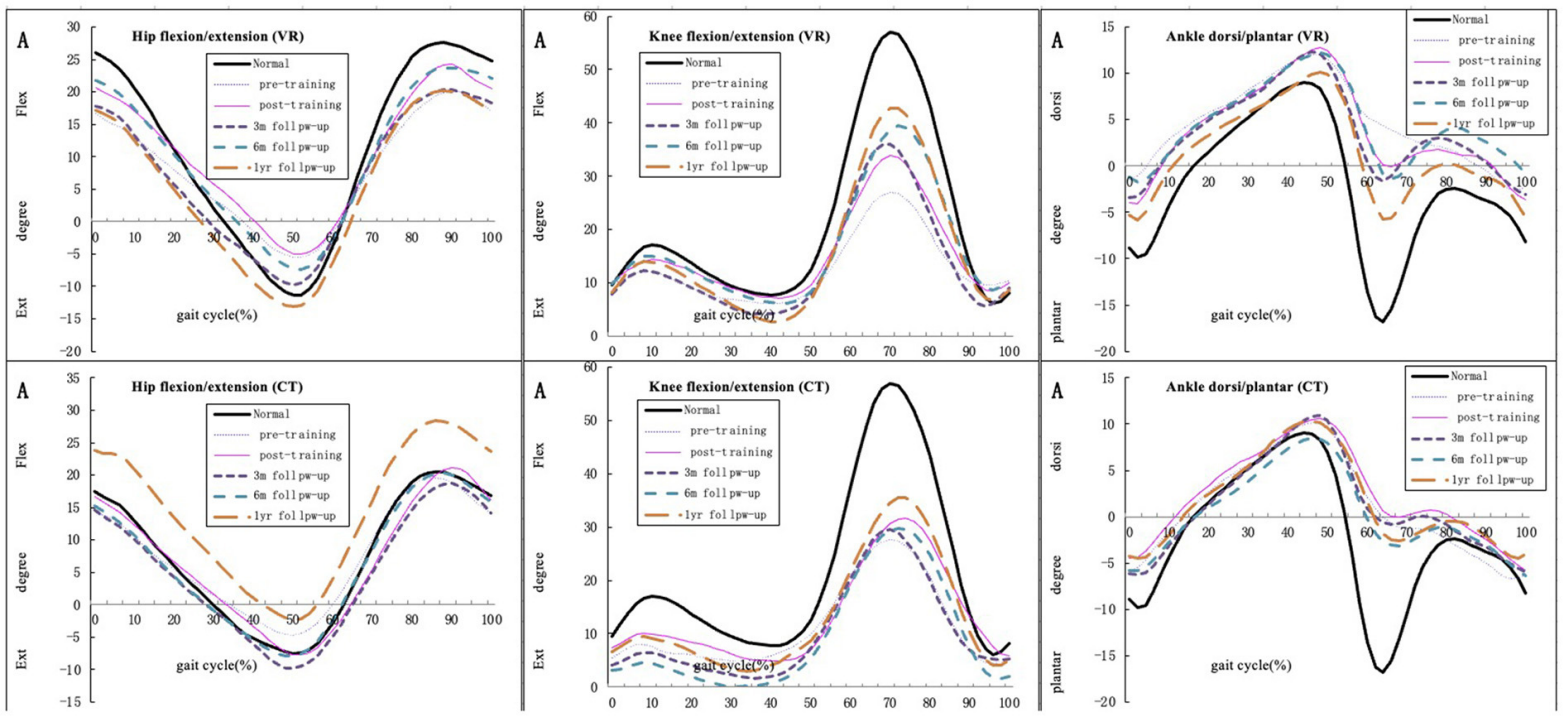
Mean hip, knee, and ankle joint kinematic curve for all participants in the sagittal plane of both groups during pre-, post-training and follow-up (compared with normal gait cycles).

Figure 3 demonstrates the mean kinematic curves for the hip, knee and ankle joints in the sagittal plane for all participants of both groups at baseline, immediately post-intervention and follow-ups.

## Discussion

The objective of this study was to determine whether non-immersive VR-based training can improve motor function and gait capability in patients with post-stroke hemiparesis and whether its short- and long-term effectiveness in enhancing lower-limb motion function recovery is greater than that of CT. Overall, our results demonstrated that VR-based training improved lower-limb motion function and gait performance, but non-immersive VR techniques were not more effective than CT techniques.

The outcome of balance capacity evaluated based on BBS scores was improved immediately after the intervention and at 3-month, 6-month, and 1-year follow-ups in both the VR and CT groups, with no significant changes between two groups at any time point. This result is consistent with previous studies that demonstrated that VR intervention is effective in improving balance performance in post-stroke patients both immediately post-intervention and at follow-ups (28, 29, 41). However, another study that adopted videogame-based training for post-stroke patients showed no significant improvement in BBS scores (32). These results indicate the importance of therapists' assistance and selecting the appropriate type of intervention. According to the FMA-LE results, lower limb motor function was improved only at the 3-month, 6-month, and 1-year follow-ups in the CT group, while VR group's outcome was improved immediately after the intervention and at 3-month, 6-month, and 1-year follow-ups; in addition, no significant difference between two groups. This finding is in accordance with previous studies that have reported immediate improvement in lower limb motor function after VR-based training (27, 28). One review concluded that VR-based therapies do not contribute to improvements in outcomes, especially when the interventions last for <3 weeks and VR is non-immersive (42). However, non-immersive VR in our study still contributed to the improvement of lower-extrenity motor performance after intervention. Some authors have pointed out that the FMA-LE only indicates abnormal synergistic motor patterns in voluntary and isolated movement tasks and is therefore insufficient to capture all of the necessary information about complicated walking performance, as walking is cyclical and involves considerable sensorimotor integration (43). The hip and knee range of motion increased in our study due to appeared isolated movement, including improvement of hip extention and knee flexion and decreased abnormal joint movement such as knee over-extention. This scale is also not sensitive enough to detect minor changes in physical function, which may have led to our finding of no differences between the VR and CT groups.

At the follow-ups, significant improvements compared with the baseline were observed in some gait parameters in the VR group. The parameters of walking speed, cadence, stride time, opposite foot contact, step time, double support, stride length and step length changed significantly from baseline to the follow-ups in the VR group, but not in the CT group; in addition, the inter-group differences were also not significant at these time points. This suggests that although the improvements in these parameters may have arisen due to recovery, VR-based training tended to contribute more to the improvements than CT. Our results on walking speed and cadence are consistent with those reported previously. In previous studies, the increases in walking speed, which is regarded as an important indicator of gait performance in post-stroke patients, were significantly greater in the VR groups than in the CT groups (15, 44–46). Another study found that walking speed changed significantly after VR intervention in participants with mild stroke than in those with moderate and severe stroke (28). The findings of our study could also be attributable to the participants learning compensatory strategies while getting accustomed to using a VR system. Allen et al. (47) also emphasized this possibility and demonstrated that participants tended to self-select more effective walking speeds by adopting compensatory strategies with the non-paretic limb. Another possible explanation for no significant differences of spatiotemporal gait parameters is that the sample size of the VR group was smaller than that of the control group due to randomized allocation and drop-out of the participants. Studies have shown that improvement in dynamic balance capability is associated with velocity and cadence (48), while lower extremity dysfunction is highly correlated with walking speed (49). In addition, step length is affected by forward propulsion, which is generated by the stance leg to enable the trunk to move forward with dynamic balance control (47). Another possible explanation for the non-significant improvements after VR-based training in our study is that the VR environment we adopted was not ecologically valid enough to be more effective than CT. Multiple studies have highlighted the technical limitation that the majority of current VR-based rehabilitation systems do not provide users with a realistic environment or real-life situations (5, 50). The VR tasks are more like simple games rather than real-world scenarios, which affects rehabilitation effectiveness and creates a potential gap between training and actual daily function.

Nevertheless, some of the kinematic parameters, especially the maximum knee joint angle in the sagittal plane, were significantly improved from baseline to 6-month follow-ups in the VR group. Though kinematic performance of hip joint had no significant difference from baseline, our study found the hip extention has been increased at follow-ups. The performance of hip and knee joint tends to a more fluent and normal gait pattern instead of stiff knee and hip pattern after the intervention in the VR group. These results suggest that VR-based training enhances knee flexion and decreases knee overextension. A study suggested that VR can improve knee strength and performance, and lower limb motor control is highly associated with balance and gait capability (51). Taken together, improvement in knee motion contributes to improved balance capacity, which is reflected in the BBS scores. Simonsen (52) pointed out that improved joint performance in the sagittal plane is correlated with enhanced walking speed, which is consistent with the findings of our study. Similarly, the possible reason for the negative result is that VR training in our study was non-immersive, which may have reduced the participants' concentration and training effectiveness. Despite some evidence supporting the benefits of an early exercise program on functional recovery, evidence supporting the use of early VR interventions to enhance functional recovery is still lacking (14).

The mechanism of performance improvements induced by VR-based training may involve neuroplasticity, motivation and high training intensity. VR is reported to enhance post-stroke experience-dependent neuroplasticity and motor learning by activating related brain regions, inducing cortical reorganization and strengthening the mirror neuron system involved in motor planning, learning and execution (25). VR also increases training motivation and engagement by reducing the perception of exertion, contributing to effortless and sustained exercise (50, 53, 54). The intrinsic and extrinsic feedback on performance and progress given by VR programs can reinforce patients' correct behavior and help maintain their level of action (25). Meanwhile, user-dependent tasks, objective progression and repetitive training, all of which are part of VR-based training, play important roles in promoting motor learning strategies in clinical practice (11). The augmented feedback from a VR-based rehabilitation system has been shown to benefit participants by enhancing their learning rate and training the mirror neuron system (50, 55).

Our study has several limitations. First, the duration of intervention in our study was not sufficient, as an intervention of at least 8 weeks is required to observe notable effects of VR-based training due to physical adaptation (18). Second, the calibration of the VR games' difficulty was not accurate in our study and acceptability of VR was not tested among patients, which may have led to poor methodological quality and lack of a clear rationale for the intervention program, particularly in terms of treatment intensity, personalized training and task variation. Third, the sample size in this study is small, so we will conduct a further study with a larger sample size. Lastly, our study analyzed only some kinematic parameters and two clinical scales, but no kinetic parameters, cognitive or physiological changes with clinical scales. Further indexes and instruments such as the hip flexor index and gait deviation index could be used to measure gait patterns (56).

In conclusion, our current study findings using non-immersive VR to demonstrate that non-immersive VR-based training improves balance and gait performance among subacute stroke patients and contributes to normal gait pattern appearance, but the effectiveness of non-immersive VR techniques is not superior to CT-based training for balance and motor function. Non-immersive VR-based training could be applied as a clinical rehabilitation therapy as well as conventional therapy.

## Trial status

The trial is still ongoing both in the Seventh Affiliated Hospital, Sun Yat-sen University and the First Affiliated Hospital, Sun Yat-sen University. The first participant was included in October 2019 and the patient recruitment in this study was completed in June 2022.

## Data availability statement

The raw data supporting the conclusions of this article will be made available by the authors, without undue reservation.

## Ethics statement

The studies involving human participants were reviewed and approved by the Ethics Committee of the Seventh Affiliated Hospital of Sun Yat-sen University and the Ethics Committee of the First Affiliated Hospital of Sun Yat-sen University. The patients/participants provided their written informed consent to participate in this study. Written informed consent was obtained from the individual(s) for the publication of any identifiable images or data included in this article.

## Author contributions

YM, DH, and MB designed the study. MB and YS recruited the participants. YW provided training instruction. YM, MB, and YS captured the gait data and three-dimensional data. YH and LW assessed the clinical scales. MB, YM, YS, and SH interpreted the data and drafted the manuscript. All authors read and approved the final manuscript.

## Funding

This research is funded by the National Key Research and Development Program of China (2020YFC2004300 and 2020YFC2004304) and 5010 Planning Project of Sun Yat-sen University of China (Grant No. 2014001).

## Conflict of interest

The authors declare that the research was conducted in the absence of any commercial or financial relationships that could be construed as a potential conflict of interest.

## Publisher's note

All claims expressed in this article are solely those of the authors and do not necessarily represent those of their affiliated organizations, or those of the publisher, the editors and the reviewers. Any product that may be evaluated in this article, or claim that may be made by its manufacturer, is not guaranteed or endorsed by the publisher.

## Abbreviations

VR: virtual reality
CT: conventional therapy
FMA: fugl-meyer assessment
BBS: berg balance scale
NHISS national institute of health stroke scale
MMSE: mini mental state examination.
